# Study on the mechanical properties and microscopic evolution mechanisms of weathered granite soil

**DOI:** 10.1038/s41598-024-75092-y

**Published:** 2024-10-17

**Authors:** Yizhao Wang, Ruiling Jia, Yadong Li, Kezheng Yang, Jie Cui, Yi Shan

**Affiliations:** 1 Guangzhou Metro Design Research Institute Co, Ltd, Guangzhou, 510620 China; 2 School of Civil Engineering and Transportation, Guangzhou University, Guangzhou, 510006 China

**Keywords:** Weathered granite soil, Weathering process, Mechanical properties, Microscopic evolution, Weathering indices, Civil engineering, Engineering

## Abstract

Studying the effects of weathering on the mechanical properties and microscopic evolution of weathered granite soil (WGS) is essential for connecting microstructure with macroscopic behavior. This study conducts systematic monotonic and cyclic triaxial tests, along with a series of microscopic tests on WGS samples, to explore the influence of weathering on WGS mechanical properties and the mechanism of granite weathering. Results indicate that both effective internal friction angle and effective cohesion decrease progressively with increased weathering. Completely weathered granite (CWG) exhibits greater dynamic strength compared to granite residual soil (GRS). Additionally, as weathering progresses, quartz fragments are lost, while feldspar and biotite weather to form secondary minerals such as kaolinite and illite, leading to an overall enrichment in aluminum and iron in the granite. Weathering causes structural deterioration of WGS. Finally, the mechanical parameters of WGS and their chemical weathering indices show a coefficient of determination ranging from 60 to 99%. This study helps elucidate the fundamental causes of performance changes in WGS, thereby optimizing engineering design and enhancing disaster prediction accuracy, while providing new research perspectives and experimental evidence for WGS.

## Introduction

Granite is widely distributed in the southern and southeastern regions of China^1^, particularly in Guangdong and Fujian provinces, and after undergoing weathering, granite forms weathered granite soil (WGS). According to the degree of weathering, it is generally classified into six levels, fresh granite, slightly weathered granite, moderately weathered granite, highly weathered granite, completely weathered granite (CWG)and granite residual soil (GRS). It is generally acknowledged that within the weathering products of granite, only granite residual soil and completely weathered granite are considered as soil materials, possessing soil characteristics^2,3^. As a type of soil that remains in place after the weathering of granite, this type of soil typically exhibits adverse engineering characteristics such as susceptibility to disturbance^4^, softening when wetted^5,6^, susceptibility to disintegration^7,8^, high porosity^4^, and poor stability^9^. WGS serves as the main load-bearing layer in engineering structures in southeastern China and finds widespread application in road construction, building construction, and slope protection engineering^10–14^. However, its application can sometimes lead to engineering hazards such as slope collapse^15,16^ and surface subsidence^17^.Therefore, research on the mechanical properties and microscopic mechanisms of WGS, including the study of factors influencing its weathering process, has significant implications for its engineering applications.

In recent years, there has been significant research on the static and dynamic properties of WGS. Studies have shown that under static loading, the static performance of GRS generally deviates from that of CWG, with its internal friction angle and cohesion decreasing with increasing weathering degree^12^. Additionally, researchers have investigated the effects of factors such as moisture content^18^, structural characteristics^19^and number of loading cycles^4^ on static performance. On the other hand, under dynamic loading, various influencing factors such as confining pressure^20^, moisture content^18^, cyclic shear amplitude^12^, and initial static deviator stress^12^ have been studied. However, previous research on the mechanical properties of CWG and GRS has mainly focused on a single type of soil, without comparing the differences in mechanical properties caused by differences in weathering degree. Moreover, there has been relatively less research on dynamic strength. Therefore, comprehensive and in-depth studies are needed to simultaneously investigate the static properties and dynamic strength properties of GRS and CWG to clarify the influence of weathering degree.

The weathering process has been extensively studied since the early 20th century, as it constitutes a fundamental component of global elemental cycling by chemically decomposing thermodynamically unstable minerals on the Earth’s surface^21^. In humid tropical and subtropical regions such as Hong Kong and Guangzhou in China, weathered granite and WGS are widespread, indicating the ubiquitous presence of weathering processes^22^. To objectively and accurately characterize weathering processes, researchers have proposed several chemical weathering indices for quantifying weathering effects. Table 1 in this paper presents the chemical weathering indices studied for WGS. These chemical weathering indices are derived from microscopic chemical composition analyses, indicating the necessity of employing microscopic research methods as auxiliary tools in weathering studies.

X-ray diffraction (XRD), X-ray fluorescence spectroscopy (XRF), and scanning electron microscopy (SEM) are widely used to detect the microscopic characteristics of soil^23^, which are considered key factors influencing and determining its engineering geological properties^19,24,25^. It is well known that changes in chemical composition and mineral composition lead to variations in the microscopic structure of soil, further affecting its macroscopic mechanical behavior^26,27^. For example, the content of iron oxides affects the degree of cementation in WGS^28^, which in turn plays a crucial role in the stiffness of the soil mass^29^. Additionally, differences in minerals and microscopic structures of WGS lead to variations in porosity, dry density, and particle strength, resulting in different mechanical characteristics and affecting its engineering properties^30,31^. Therefore, the microscopic evolution caused by chemical weathering is closely related to the macroscopic mechanical characteristics. Previous studies have investigated the relationship between chemical weathering indices and physical indices^32–34^. Building upon this foundation, Chiu^26^ studied the relationship between chemical weathering indices and the shear strength and dilation of granitic saprolites. Liu^35^ further expanded the experimental scope to evaluate the applicability of relevant chemical weathering indices to granite weathering layers. However, the current understanding of the weathering evolution of granite remains incomplete, particularly regarding the transition from fresh granite (FG) to CWG and then to GRS. Therefore, it is necessary to study the mineral composition, chemical composition, and microscopic structure of FG and WGS to analyze the microscopic mechanisms of granite weathering evolution. Furthermore, there is still insufficient research on the differences in mechanical properties of WGS induced by weathering processes, and there is a lack of understanding of the connection between microscopic mechanisms and mechanical properties. Hence, there is a need to quantitatively establish weathering indices for WGS and develop empirical models correlating these indices with mechanical parameters.

In light of the above background, this paper focuses on WGS formed during the Yanshan period. It investigates the static and dynamic mechanical properties of WGS through consolidated undrained triaxial tests, and analyzes the microscopic evolution mechanism from bedrock to WGS using XRD, XRF, and SEM. Additionally, it establishes the relationship between chemical weathering indices and mechanical parameters. This study contributes to a better understanding of chemical weathering in weathered granite and its relationship with the mechanical properties of weathering layers.

**Table 1:** Comprehensive table of weathering indices.

Number	Index name	Source of index	Formula
1	*I*_mob_^32^:, mobiles index	Irfan (1996)	$$\begin{gathered} \left( {{I_{{\text{fresh}}}} - {I_{{\text{weathered}}}}} \right){\text{ }}/{\text{ }}{I_{{\text{fresh}}}}, \hfill \\ I{\text{ }}={\text{ }}\left( {{{\text{K}}_2}{\text{O}}+{\text{N}}{{\text{a}}_2}{\text{O}}+{\text{CaO}}} \right) \hfill \\ \end{gathered}$$
2	MWPI^36^, Modified Weathering Potential Index	Vogel (1975)	$$\begin{gathered} {\text{100\% }} \times \left( {{{\text{K}}_{\text{2}}}{\text{O + N}}{{\text{a}}_{\text{2}}}{\text{O + CaO + MgO}}} \right){\text{ }} \hfill \\ {\text{/ (Si}}{{\text{O}}_{\text{2}}}{\text{ + A}}{{\text{l}}_{\text{2}}}{{\text{O}}_{\text{3}}}{\text{+F}}{{\text{e}}_{\text{2}}}{{\text{O}}_{\text{3}}}{\text{ + Ti}}{{\text{O}}_{\text{2}}} \hfill \\ {\text{ + CaO + N}}{{\text{a}}_{\text{2}}}{\text{O + MgO + }}{{\text{K}}_{\text{2}}}{\text{O)}} \hfill \\ \end{gathered} $$
3	Ba^37^, B-series index	Rocha (1985)	$\left( {{{\text{K}}_{\text{2}}}{\text{O + N}}{{\text{a}}_{\text{2}}}{\text{O + CaO}}} \right){\text{ / A}}{{\text{l}}_{\text{2}}}{{\text{O}}_{\text{3}}}$
4	ba_1_^37^_,_ B-series index	Rocha (1985)	$\left( {{\text{N}}{{\text{a}}_{\text{2}}}{\text{O + }}{{\text{K}}_{\text{2}}}{\text{O}}} \right){\text{ / A}}{{\text{l}}_{\text{2}}}{{\text{O}}_{\text{3}}}$
5	Base: alumina^38^, Substrate oxidation index	Colman (1982)	$\left( {{{\text{K}}_{\text{2}}}{\text{O + N}}{{\text{a}}_{\text{2}}}{\text{O + CaO + MgO}}} \right){\text{ / A}}{{\text{l}}_{\text{2}}}{{\text{O}}_{\text{3}}}$
6	IOL^21^, Index of laterization	Babechuk (2014)	$$\begin{gathered} \left( {{\text{A}}{{\text{l}}_{\text{2}}}{{\text{O}}_{\text{3}}}{\text{ + F}}{{\text{e}}_{\text{2}}}{{\text{O}}_{\text{3}}}} \right) \times {\text{100\% }} \hfill \\ {\text{ / }}\left( {{\text{A}}{{\text{l}}_{\text{2}}}{{\text{O}}_{\text{3}}}{\text{ + F}}{{\text{e}}_{\text{2}}}{{\text{O}}_{\text{3}}}{\text{ + S}}{{\text{i}}_{\text{2}}}{\text{O}}} \right) \hfill \\ \end{gathered} $$

## Materials and methods

### Triaxial test scheme

**Fig. 1:**
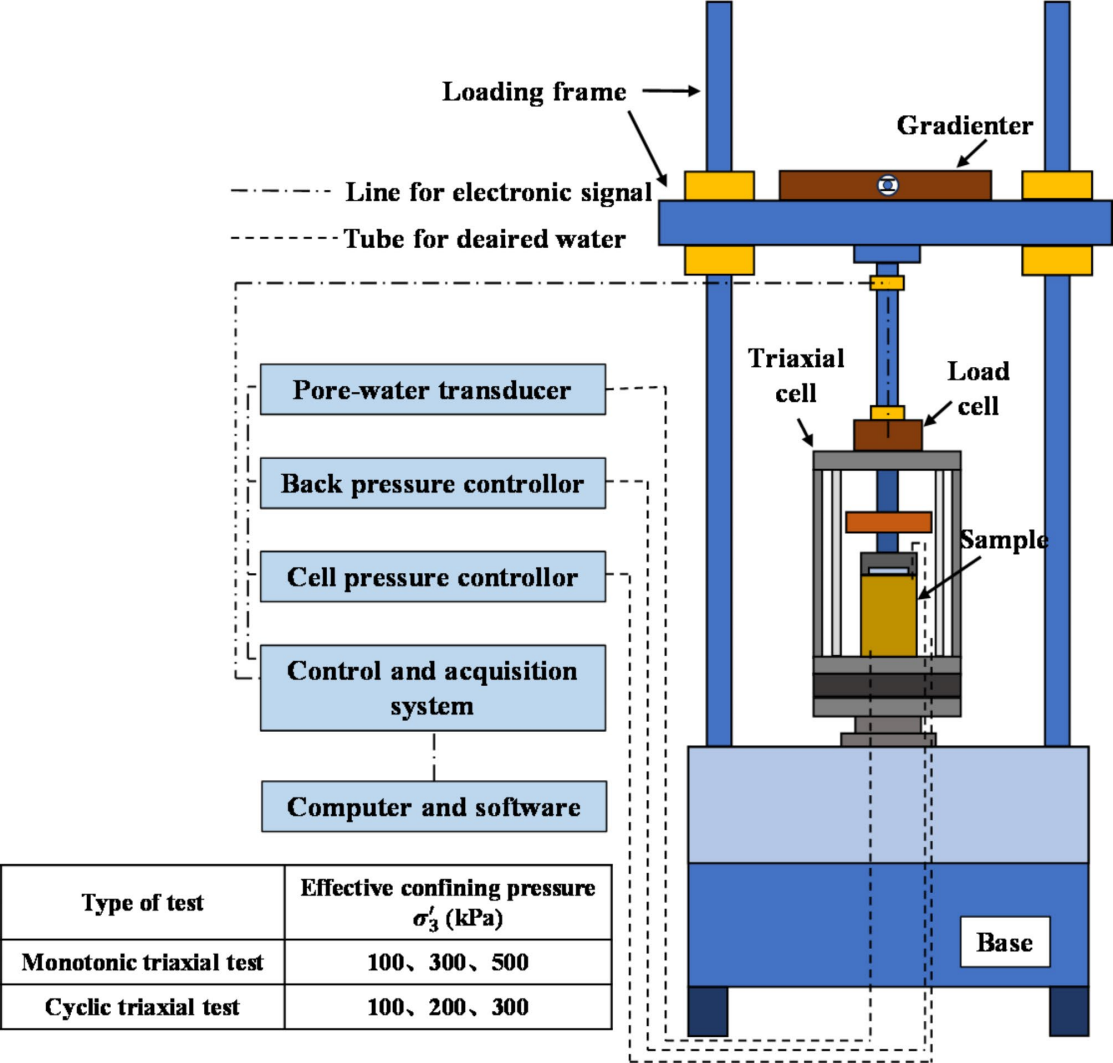
Schematic diagram of adopted triaxial test setup.

In this test, the static and dynamic mechanical properties of WGS were studied using static and dynamic triaxial instruments. The testing principles of the instruments are identical, and they are composed of five parts: a pore-water transducer, a back pressure controller, a cell pressure controller, a control and acquisition system, and a computer with software. During the experiment, the chamber is filled with water. The controller applies confining pressure to the specimen, and axial force is applied to the specimen through an axial rod. The specimen dimensions are 39.1 mm × 80 mm. The pressure controller can reach a maximum of 2 MPa, and the maximum vibration frequency is 10 Hz. A schematic diagram of the instrument is shown in Fig. 1.

Monotonic triaxial tests were conducted using a TAS-LF static triaxial testing machine to perform a series of consolidated undrained monotonic triaxial tests on weathered granite samples with different degrees of weathering. The test procedure begins with saturation using the vacuum saturation method, followed by water head saturation and back-pressure saturation on a triaxial apparatus, ensuring that the pore pressure coefficient (B ≥ 0.95). Subsequently, consolidation treatment is applied before loading. The shear strain rate was controlled at 0.02 mm/min, and the test was terminated when significant failure of the specimen was observed or axial strain reached 15%^39^.

Cyclic triaxial tests were conducted using a DDS triaxial testing machine to perform consolidated undrained cyclic tests on soil samples. The loading method for dynamic loading was cyclic loading, with a sinusoidal waveform at a frequency of 1 Hz. The pre-treatment steps for the specimen before the test loading are identical to the aforementioned procedures for static triaxial tests. The test was terminated when the strain exceeded 1% or when the pore water pressure equaled the confining pressure.

Overall, this study for the GRS and CWG samples from boreholes B1 and B2, monotonic triaxial tests were conducted under effective confining pressures of 100, 300, and 500 kPa, respectively, along with cyclic triaxial tests conducted under effective confining pressures of 100, 200, and 300 kPa.

### **Microscopic test scheme**

To investigate the microscopic evolution mechanisms of WGS, this study conducted XRD, XRF, and SEM on both the FG and WGS. XRD was employed to determine the mineral composition. Samples were dried, ground into powder, and sieved for analysis. The sample was scanned by electron microscopy using the Rigaku Ultima IV instrument made in Japan, with the scanning speed set at 5°/min and the scanning range from 10° to 80°. XRD data analysis, including peak extraction and phase identification, was conducted using MDI Jade 9 software. XRF analysis was conducted using the RIGAKU ZSX Primus model instrument. Appropriate X-ray energy levels were selected based on the energy levels of the elements to be tested and the excitation method, ensuring clear fluorescence peaks and accurate analytical results. XRF was employed to determine the chemical composition of both the fresh granite and its weathered soil samples. The microscopic structure of weathered granite was analyzed via SEM. The dry specimens for SEM examination were prepared via vacuum freeze–drying technique to minimize changes of soil microstructure caused by shrinkage.

### Materials and samples

**Fig. 2:**
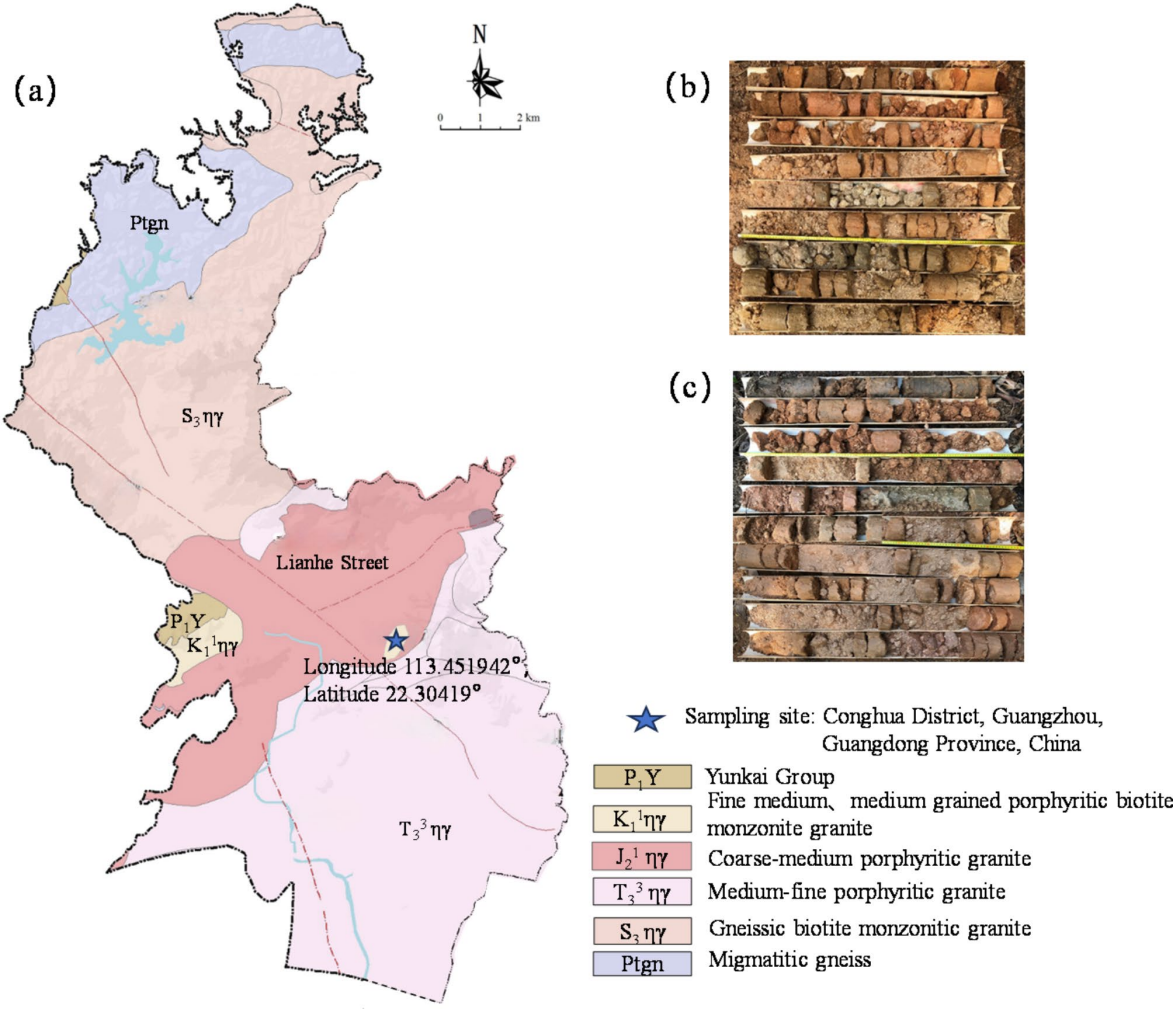
Sampling location geological section of the present study site: (**a**) Bedrock geological map of Lianhe Street, Huangpu district, the authors used ArcGIS software (Ver. 10.8) (https://desktop.arcgis.com/zh-cn/quick-startguides/10.8/) to perform the map. (**b**) the stratigraphic section of the sampling site B1, (**c**) the stratigraphic section of the sampling site B2.

**Fig. 3:**
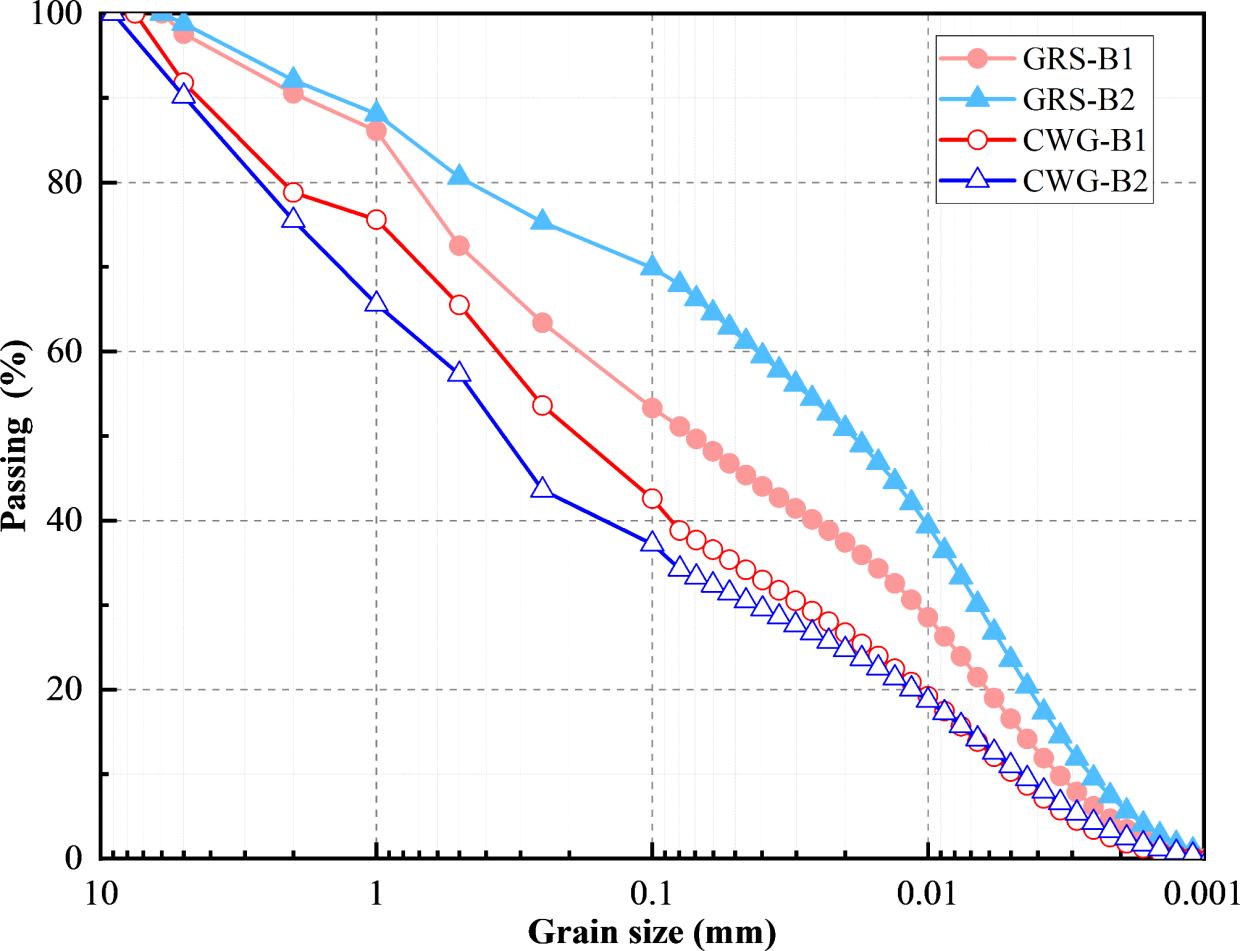
Particle size distribution curve.

The WGS’s samples used in the experiments were collected from a slope at a construction site in the Huangpu District of Guangzhou City, as shown in Fig. 2. The bedrock of the slope mainly consists of Yanshan period I porphyritic biotite monzogranite, with mineral compositions primarily including plagioclase, biotite, and quartz. The WGS is derived from the weathering of the biotite monzogranite, which tends to soften and disintegrate upon contact with water. Soil samples were obtained from two different locations, boreholes B1 and B2, using a drilling machine. GRS and CWG soil samples were taken for testing. According to the specifications outlined in Code for Investigation of Geotechnical Engineering^40^ and ISRM^41^ standards, standard penetration tests (SPT) were conducted to differentiate between GRS and CWG. The SPT is an in-situ testing method used to measure the density of soils, which provides empirical estimates of the stiffness and strength parameters of soil deposits and weathered layers beneath the bedrock stratum^42^. The standard penetration blow counts and their corrected values for WGS obtained in this test are presented in Table 2. Laboratory tests were conducted on the soil samples in accordance with Chinese standard for geotechnical testing method^43^. The summarized properties of the various parameters are presented in Table 2. Figure 3 shows the cumulative particle size distribution curve of WGS. Combining the parameters from Table 2, it is evident that the WGS exhibits a relatively high coefficient of uniformity, and the average particle size *D*_50_ decreases with increasing weathering degree. Moreover, in the GRS, the proportion of particles with sizes below 0.075 mm is 50% for borehole B1 and 68% for borehole B2, primarily comprising silt and clay particles. Conversely, in CWG, the proportion of particles with sizes below 0.075 mm is only 38% for borehole B1 and 33% for borehole B2, with the soil containing a higher proportion of sand particles in addition to silt and clay.

**Table 2:** The basic physical and mechanical parameters of WGS.

Sample	GRSB1	CWGB1	GRSB2	CWGB2
Depth (m)	6.3–6.6	7.7-8.0	1.0-1.5	8.7-9.0
Natural density *ρ* (g/cm^3^)	1.96	2.06	1.97	2.02
Dry density *ρ*_d_ (g/cm^3^)	1.56	1.74	1.59	1.68
Water content *w* (%)	25.61	18.55	23.68	19.69
Specific gravity *G*_s_	2.67	2.68	2.66	2.61
Void ratio *e*	0.71	0.54	0.67	0.55
Liquid limit *w*_L_	45.89	42.40	44.61	42.33
Plastic limit *w*_p_	28.19	28.26	24.15	28.83
Plasticity index *I*_p_	17.70	14.40	20.45	13.50
*D*_50_ (mm)	0.071	0.201	0.019	0.367
*C*_u_	39.3	51.9	16.6	139.1
Color	Red brown	Yellow	Red brown	Yellow brown
SPT-*N*	27	40	23	48
SPT-$\:{N}^{{\prime\:}}$	25.9	37	22.1	44.4

## Test results analysis

### Monotonic Triaxial tests

**Fig. 4:**
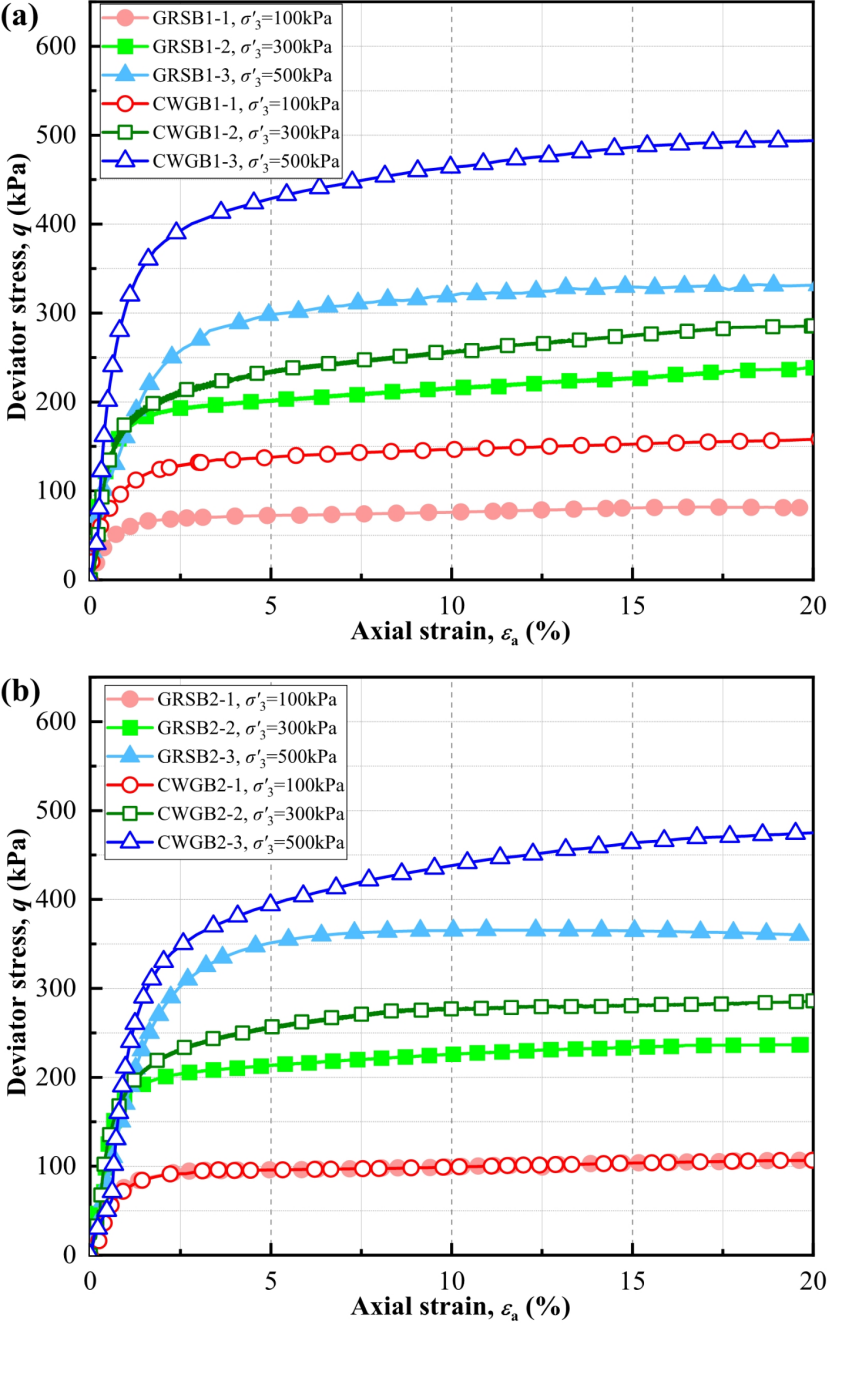
Development of deviatoric stress versus axial strain for each specimen in monotonic test. (**a**) B1; (**b**) B2.

This section analyzes and explores the results of the monotonic triaxial tests on WGS. Figure 4 illustrates the variation of deviator stress (*q*) with axial strain $\it \:{\epsilon\:}_{a}\:$for each specimen, showing that the stress-strain curves of the WGS specimens exhibit continuous hardening behavior. The *q* increases rapidly during the initial loading stage, then the rate of increase diminishes when the axial strain reaches approximately 2.5%. Subsequently, the *q* tends to stabilize with increasing axial strain. Moreover, the CWG at the same borehole exhibits a higher initial modulus and a greater peak stress compared to the GRS.

According to the Coulomb equation, the strength envelope of soil can be expressed as:

1 $$\:{\tau\:}_{f}={\sigma\:}^{{\prime\:}}{tan}{\phi\:}^{{\prime\:}}+{c}^{{\prime\:}}$$

**Fig. 5:**
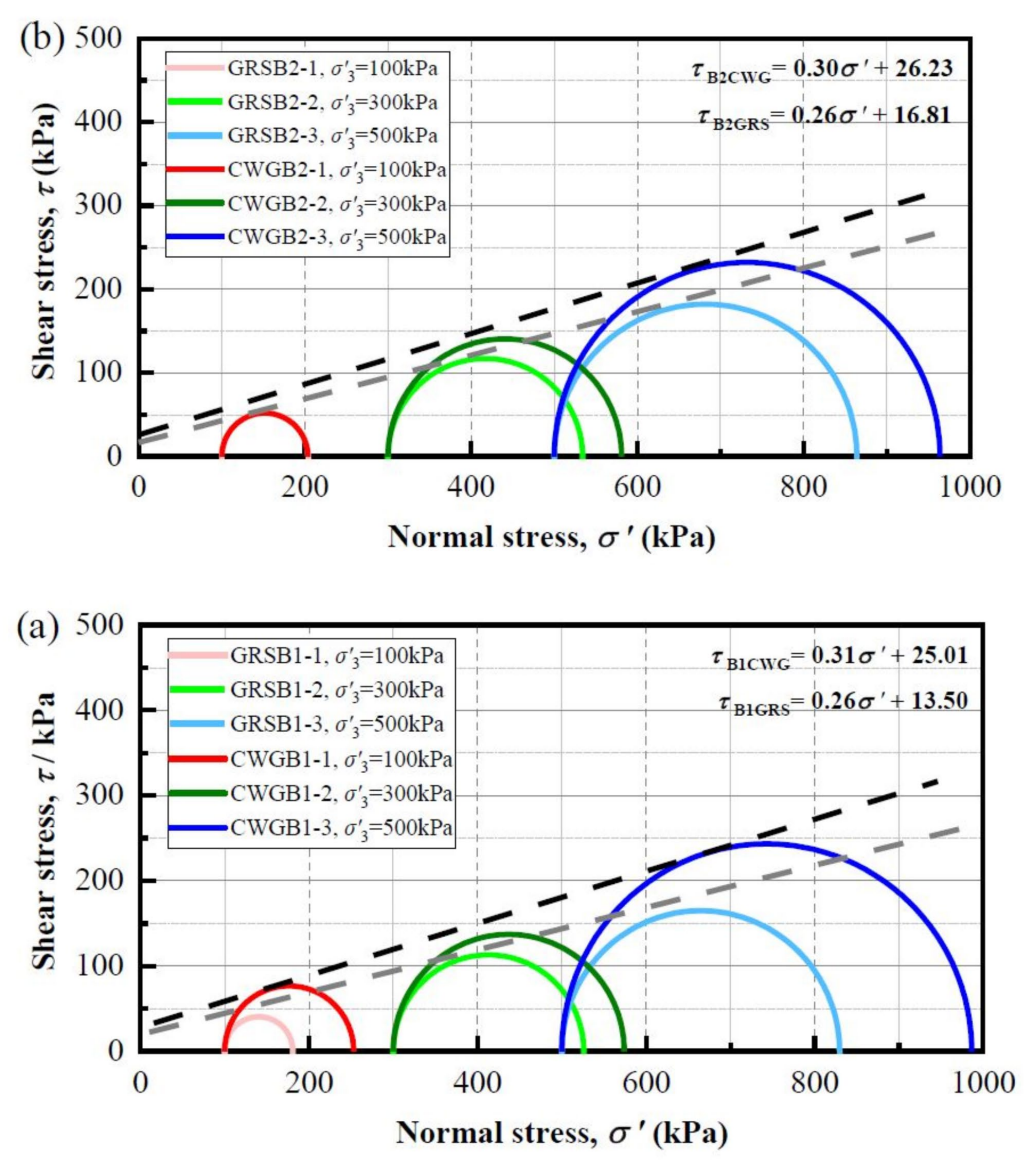
Mohr stress circle and strength envelope of WGS. (**a**) B1; (**b**) B2.

In the equation: $\:{\tau\:}_{f}\:$represents the shear strength of the soil; $\:{c}^{{\prime\:}}$ denotes the effective cohesion of the soil; $\:{\sigma\:}^{{\prime\:}}$ signifies the effective normal stress on the shear failure plane; and $\:{\phi\:}^{{\prime\:}}$ stands for the effective internal friction angle of the soil. Since the stress-strain curves of the WGS samples in the triaxial tests exhibit continuous hardening behavior and lack a peak, the *q* corresponding to an axial strain of 15% is taken as the peak stress. With the peak stress and effective confining pressure, the Mohr circles and envelope lines of shear strength are plotted according to the Mohr-Coulomb criterion. Figure 5 illustrates the Mohr stress circle and strength envelope of WGS, revealing that the effective cohesion of CWG is significantly greater than that of GRS. This can be attributed to the structural damage incurred by WGS due to weathering, resulting in weakened bonding forces and diminished cohesion. In contrast, CWG, having a lower degree of weathering compared to GRS, exhibits more stable cohesion, thus generating greater shear strength from bonding forces. It is generally believed that the main factors affecting the internal friction angle of soil include density, void ratio, particle size distribution, particle shape, and mineral composition. As evident from Fig. 5, the effective internal friction angle of CWG is greater than that of GRS. This can be attributed to CWG’s lower degree of weathering, with particles more closely resembling the fresh granite, resulting in greater differences in particle shape and easier interlocking, as well as rough and uneven mineral contact surfaces leading to increased sliding friction, thus resulting in a larger effective internal friction angle^44^. Additionally, CWG has coarser particles, greater heterogeneity, and higher density, resulting in stronger interlocking action and a larger effective internal friction angle.

### Cyclic Triaxial tests

The dynamic strength of soil is typically expressed as the dynamic stress amplitude required to cause soil failure under a specified number of cycles, or conversely, as the number of cycles required to induce soil failure under a given dynamic stress amplitude. This relationship is represented by a curve depicting the relationship between the dynamic stress amplitude $\:{\sigma\:}_{\text{d}}$ causing soil failure and the corresponding number of cycles (*N*). To mitigate the influence of variations in dynamic stress magnitude, the relationship between the cyclic stress ratio (CSR) and the number of cycles (*N*) at soil failure is commonly plotted. In this study, 1%, which is regarded as the allowable strain, represents the strain that WGS can withstand. Under this strain level, a power curve is employed to describe the relationship between CSR and N.

2 $$\:\text{C}\text{S}\text{R}\propto\:k{\text{N}}^{-b}$$

In the equation, *k* and *b* are fitting parameters, and CSR is defined as the ratio of the cyclic shear stress amplitude to twice the effective confining pressure.

**Fig. 6:**
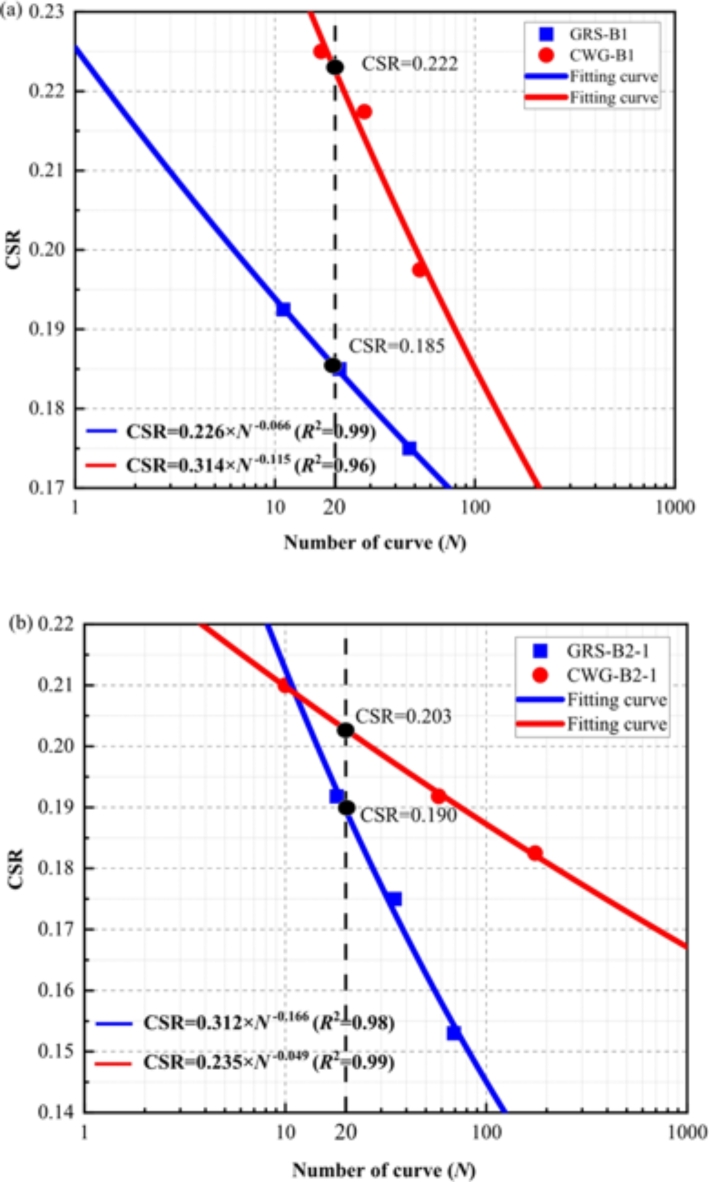
The fitting curve of the relationship between CSR-*N*. (**a**) B1; (**b**) B2.

The relationship between CSR and *N* for different soil samples is obtained according to the above formula, as shown in Fig. 6. As CSR increases, the required *N* value to reach 1% strain decreases. It is evident that with increasing CSR, the dynamic stress amplitude increases, and the dynamic strain increases more rapidly, resulting in fewer cycles required to reach the allowable strain. Based on the correspondence between seismic magnitude, seismic intensity, and equivalent number of cycles (*N*_eq_) when *τ* = 0.65*τ*_max_ given by Seed^45^, the equivalent cycles 20 corresponding to a seismic magnitude of 7.5 (*CSR*_20_) are selected as key parameters for discussion. As shown in Fig. 6, the *CSR*_20_ of CWG in both B1 and B2 holes is larger than that of GRS, indicating that CWG has greater reliability in engineering use compared to GRS. Overall, when CSR is the same, CWG requires more cycles to reach the allowable strain compared to GRS, indicating that CWG has higher dynamic strength than GRS. This is because the degree of weathering in CWG is lower than that in GRS, and CWG has a denser structure than GRS^28^. However, for the soil in hole B2, in cases where CSR is greater than 0.21, the GRS requires more cycles to reach the allowable strain compared to CWG, which is contrary to other situations and requires further investigation.

### Interpretation of microscopic observations

**Fig. 7:**
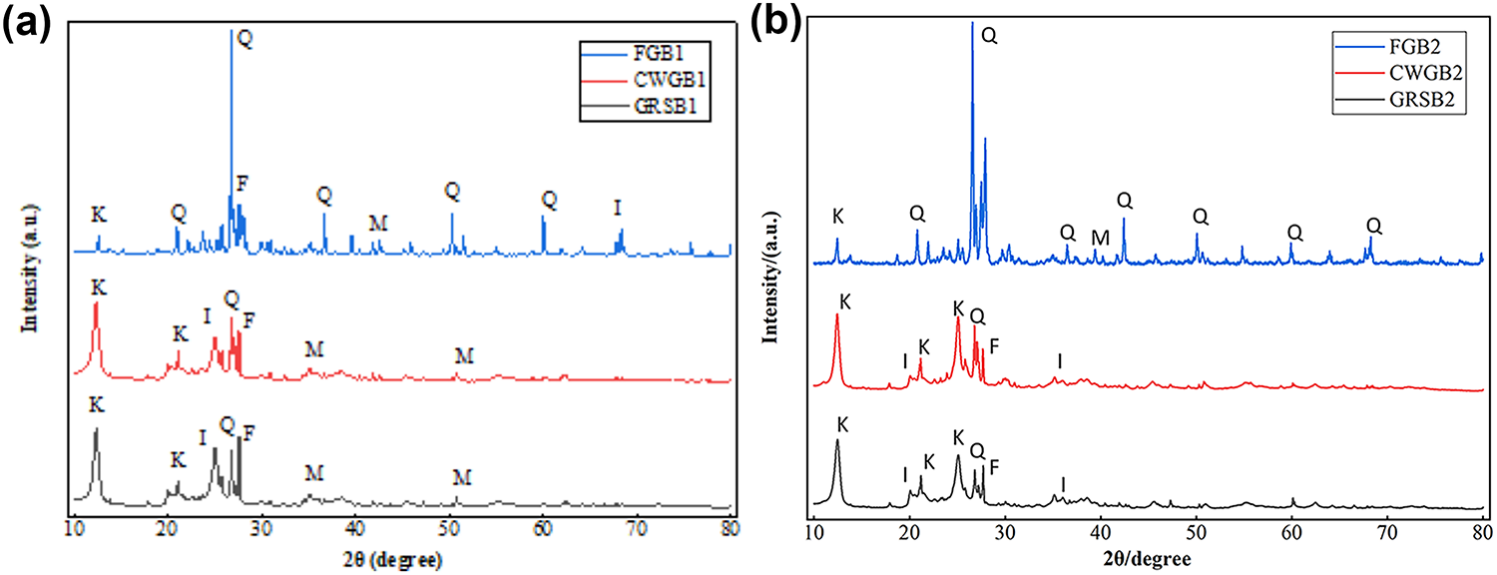
Mineral composition analysis of sample by XRD test. Note: K - Kaolinite, Q - Quartz, F - Feldspar, I - Illite, M - Mica; Due to the low content of hematite, there is no significant diffraction peak in the XRD spectra. (**a**) B1; (**b**) B2.

**Fig. 8:**
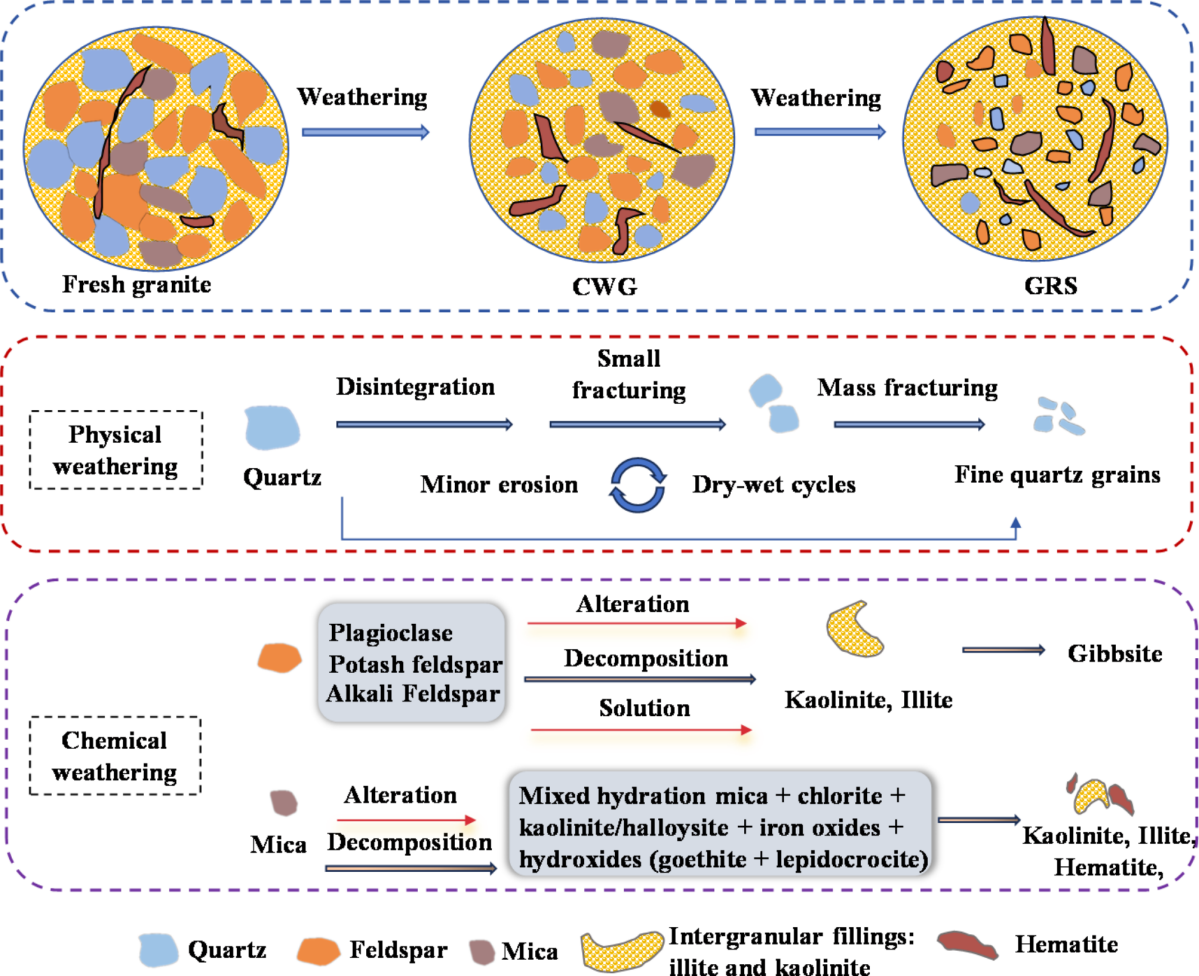
Mineral evolution diagram during granite weathering process.

In order to elucidate the microstructural effects of weathering on WGS, this study conducted XRD analysis to determine the phase composition of WGS and FG, and analyzed their main minerals and contents. The obtained XRD spectra are shown in Fig. 7, and the main minerals and their contents are presented in Table 3. XRF was employed to determine the major chemical composition of the research samples, and the test results are summarized in Table 3. XRD analysis revealed that the main minerals in FG and WGS consist of kaolinite, quartz, feldspar, illite, mica, and hematite. In FG, quartz is the dominant mineral component; however, as weathering progresses, kaolinite gradually becomes the dominant mineral, while the quartz content decreases sharply. Generally, quartz has high hardness and strong electrochemical stability, being less affected by chemical weathering or showing slight metamorphism. It mainly undergoes fragmentation of large particles into smaller ones due to weathering^35^. Feldspar and biotite particles are replaced by clay minerals and iron oxide. Overall, the early stages of chemical weathering primarily involve the hydrolysis and hydration of alkali metals (K, Na) and alkaline earth metals (Ca), accompanied by desilication, resulting in overall aluminization and ferritization. Figure 8 illustrates the mineral evolution during the granite weathering process. The chemical formula for the weathering process of WGS is as follows^46,47^:

3 $$\begin{aligned} ~ & {\text{NaAlS}}{{\text{i}}_{\text{3}}}{{\text{O}}_{\text{8}}}{\text{(Albite) + KAlS}}{{\text{i}}_{\text{3}}}{{\text{O}}_{\text{8}}}{\text{(Orthoclase) + CaA}}{{\text{l}}_{\text{2}}}{\text{S}}{{\text{i}}_{\text{2}}}{{\text{O}}_{\text{8}}}{\text{(Anorthite)}} \\ & \quad {\text{+ K}}{\left( {{\text{Mg,Fe}}} \right)_{\text{3}}}{\text{AlS}}{{\text{i}}_{\text{3}}}{{\text{O}}_{{\text{10}}}}{\left( {{\text{F,OH}}} \right)_{\text{2}}}{\text{(Biotite) }} \to {\text{ A}}{{\text{l}}_{\text{4}}}{{\text{(OH)}}_{\text{8}}}{\text{(Si}}{{\text{O}}_{{\text{10}}}}{\text{)(Kaolinite)}} \\ & \quad {\text{+ }}{{\text{K}}_{{\text{0}}{\text{0.7}}}}{\text{A}}{{\text{l}}_{\text{2}}}{{\text{(Si,Al)}}_{\text{4}}}{{\text{O}}_{{\text{10}}}}({\text{Illite}}){\text{ + F}}{{\text{e}}_{\text{2}}}{{\text{O}}_{\text{3}}}{\text{ + C}}{{\text{a}}^{2+}}{\text{ + N}}{{\text{a}}^{\text{+}}} \\ \end{aligned}$$

**Table 3:** The mineral composition and chemical constituents of WGS and FG.

Sample	Mineral composition (%)						Main chemical constituents (%)									
	Kaolinite	Quartz	Feldspar	Illite	Mica	Hematite	SiO_2_	Al_2_O_3_	Fe_2_O_3_	K_2_O	TiO_2_	MgO	*P*_2_O_5_	Na_2_O	CaO	SO_3_
GRSB1	39.4	6.9	22.4	14.7	13.3	3.3	51.73	35.65	6.99	3.50	0.98	0.84	0.14	0.096	0.056	0.013
CWGB1	36.9	7.1	23.2	16.6	13.2	3.0	52.46	34.96	6.30	3.97	0.94	1.11	0.13	0.084	0.031	0.009
GRSB2	38.7	5.0	20.3	17.2	15.1	3.7	50.91	35.89	7.31	3.61	1.19	0.74	0.19	0.062	0.044	0.047
CWGB2	37.7	8.2	22.2	16.3	12.7	2.9	51.91	35.74	5.89	3.88	1.09	1.09	0.27	0.074	0.044	0.007
FGB1	18	31	25.4	11.6	12.5	1.5	65.58	16.95	3.62	5.76	0.67	1.62	0.58	2.490	2.710	0.014
FGB2	17.6	30.3	26.7	11.8	12.0	1.6	66.68	16.35	3.33	5.63	0.72	1.52	0.55	2.665	2.544	0.011

**Fig. 9:**
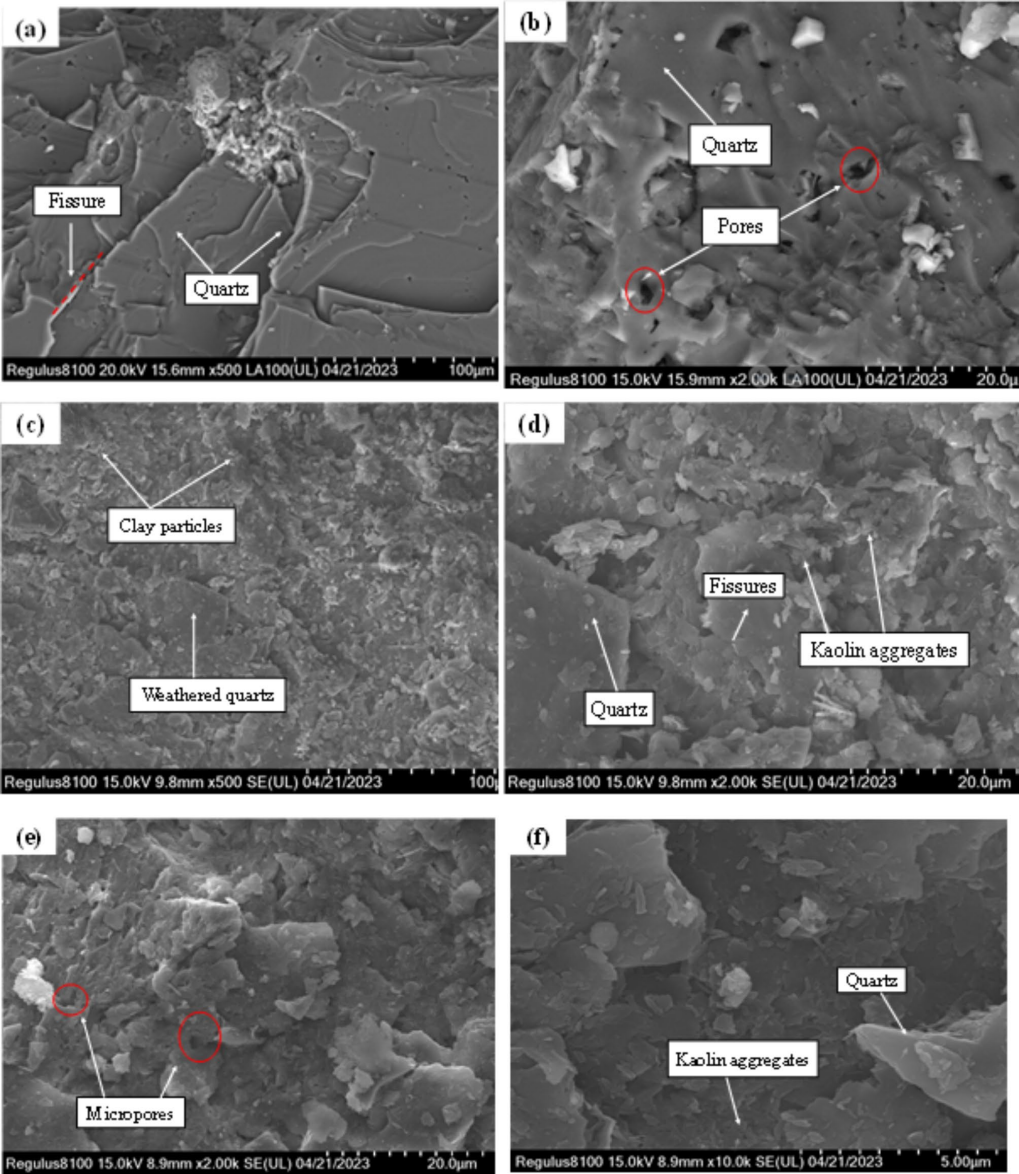
SEM images of FG and WGS. (**a**) FG magnified 500 times; (**b**) FG magnified 2000 times; (**c**) CWG magnified 500 times; (**d**) CWG magnified 2000 times; (**e**) GRS magnified 2000 times. (**f**) GRS magnified 10,000 times.

SEM images intuitively demonstrate the impact of weathering on the microstructure from the fresh granite to CWG and then to GRS. As shown in Fig. 9, the surface of the FG rock is smooth with clear textures, dense cementation, and intact structure. The mineral particles are tightly bonded, showing overall integrity, with a few scattered loose particles and rock fragments on the rock surface, accompanied by small pores and no significant cracks. From Fig. 9 (d) and Fig. 9 (f), it is evident that CWG and GRS have abundant flaky particles, with many fine particles attached to them. According to previous studies^29,48^, these fine particles consist mainly of clay minerals, predominantly kaolinite, forming loose, open aggregates bonded by point-to-face or face-to-face contacts, resulting in a relatively high porosity of CWG and GRS. Meanwhile, the flaky particles stack in a face-to-face manner, forming a soil skeleton with particles interlaced and intermixed in space. The bond between mineral particles in CWG and GRS is loose, with weathering causing mineral particle detachment, resulting in uneven surfaces, obvious weathering cracks, and relatively more fractured particles compared to FG. Consequently, as weathering progresses, the particles of FG and CWG become more dispersed, exacerbating structural deterioration.

## Discussion and comment

**Fig. 10:**
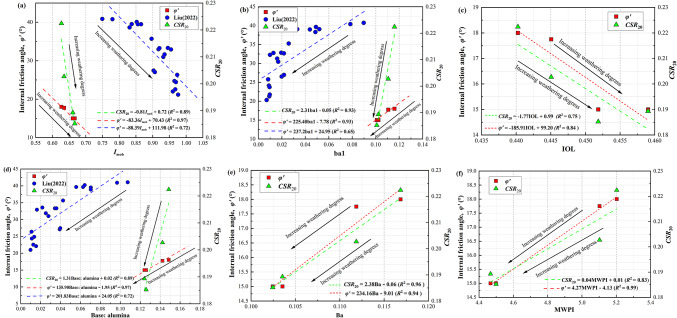
Correlation fitting between weathering indices, effective internal friction angle $\:{\phi\:}^{{\prime\:}}$, and *CSR*_20_. Partial data sources-Liu^35^ (2022). (**a**) Relationships between the *I*_mob_, $\:{\phi\:}^{{\prime\:}}$ and *CSR*_20_. (**b**) Relationships between the *ba*_1_, $\:{\phi\:}^{{\prime\:}}$ and *CSR*_20_. (**c**) Relationships between the IOL, $\:{\phi\:}^{{\prime\:}}$ and *CSR*_20_. (**d**) Relationships between the Base: alumina, $\:{\phi\:}^{{\prime\:}}$ and *CSR*_20_. (**e**) Relationships between the Ba, $\:{\phi\:}^{{\prime\:}}$ and *CSR*_20_. (**f**) Relationships between the MWPI, $\:{\phi\:}^{{\prime\:}}$ and *CSR*_20_.

From the above mechanical property tests and microscopic experiments, it is evident that different degrees of weathering lead to variations in the physical, mechanical properties, and microstructural mechanisms of WGS. Therefore, this section delves deeper into this relationship, combining the aforementioned study on the chemical weathering of WGS to quantify the weathering process into index values based on changes in chemical composition. These values are then correlated with static parameters to further investigate the relationship between chemical composition changes and static properties of GRS and CWG, while also evaluating the applicability of previous chemical weathering indices^26,35^. Additionally, based on the fitting of static parameters and chemical weathering indices, this section introduces dynamic-related parameters to study the correlation between dynamic parameters of WGS and chemical weathering indices. Table 4 lists some commonly used indicators for quantitative analysis of chemical weathering intensity. The calculated values of the weathering indices studied in this paper are listed in Table 4 based on XRF analysis. The *I*_mob_ indice serves as an indicator of the degree of feldspar decomposition. Additionally, studies have shown a positive correlation between the *I*_mob_ index and the content of fine particles^26^, both of which are significant for determining the degree of granite weathering. The Modified Weathering Potential Index (MWPI) compares the aluminum surplus with the Weathering Potential Index^49^ to measure the potential for rock weathering. The substrate oxidation index (Base: alumina) refers to the content of Al_2_O_3_ in the chemical composition of the studied rock or soil, which is related to the formation of clay minerals and aids in observing the overall process of chemical weathering. The Ba and ba_1_ components in the B-series index are primarily focused on the content of Al_2_O_3_, with Ba and ba_1_ showing a negative correlation with weathering degree. The Index of Laterization (IOL) was proposed by M.G. Babechuk^50^ to quantify the chemical changes during the late-stage chemical weathering of laterite. This index is introduced in this paper to quantify the weathering of granite, allowing for a reasonable evaluation of the degree of silicate leaching and aluminum and iron enrichment in granite, thereby understanding the strength of its weathering. In summary, these weathering indices mainly reflect the weathering of granite by focusing on changes in chemical composition during the weathering process, which further affects its microstructure and macroscopic static and dynamic characteristics. It is necessary to characterize the mechanical properties of granite and its weathered soil using weathering indices. Therefore, Fig. 10 fits the chemical weathering indices with effective internal friction angle and *CSR*_20_, respectively, visually demonstrating the variation in the mechanical properties of WGS quantified by chemical weathering indices with weathering degree. Additionally, some data from other literature are referenced for fitting. From Fig. 10, it can be observed that the variation trends between the effective internal friction angle and chemical weathering indices *I*_mob_, ba_1_, and Base: alumina in this study are consistent with those in other literature, showing a linear relationship, and the variation trend with weathering degree is also consistent. However, there are differences in the slope of the graphs fitted in this study compared to those fitted by other researchers, which may be attributed to the specific relationship between chemical weathering indices and mechanical parameters of WGS at particular locations. In most cases, these indices fit well with dynamic parameters, with correlation coefficients (*R*^2^) greater than 0.8, indicating a close relationship between chemical weathering and the evolution of mechanical properties. The fitting effect of IOL is slightly poorer, indicating that the application of weathering indices requires comprehensive consideration of the parameter configuration of selected indices and the interference of exogenous factors on the analysis results. Therefore, the integrated use of multiple indices and mutual verification is essential.

**Table 4:** The weathering indices value of WGS.

Sample	I_mob_	MWPI	Ba	ba_1_	Base: alumina	IOL
GRSB1	0.667	4.499	0.102	0.101	0.126	0.452
CWGB1	0.627	5.203	0.117	0.116	0.149	0.440
GRSB2	0.661	4.467	0.104	0.102	0.124	0.459
CWGB2	0.635	5.102	0.112	0.111	0.142	0.445
FGB1	0	12.656	0.647	0.487	0.742	0.239
FGB2	0	12.428	0.662	0.507	0.755	0.228

## Conclusions

The present study conducted monotonic triaxial tests, cyclic triaxial tests, and a series of microscopic experiments on WGS. The main conclusions are as follows:

1. Under static loading, the effective cohesion of CWG in boreholes B1 and B2 is 26.3° and 27.54°, higher than GRS’s 13.98° and 17.4°. Similarly, CWG has a larger effective internal friction angle than GRS. This is due to CWG’s lower weathering degree, leading to greater shear strength from a stronger particle skeleton. CWG’s larger particle size, higher density, better interlocking, and stronger friction also contribute. Under cyclic loading, weathering affects CWG’s dynamic properties. Its denser structure gives it greater resistance to deformation, requiring more cycles to reach the allowable strain.
2. With increasing weathering degree from fresh FG to CWG to GRS, quartz undergoes fragmentation and loss, while feldspar and biotite weather to form secondary minerals such as kaolinite and illite. The SiO_2_ content decreases, while the Al_2_O_3_ and Fe_2_O_3_ content increases. Na and Ca content are nearly depleted, while Fe content notably increases. The SEM results indicate that weathering leads to the deterioration of soil structure, dispersion of mineral particles, and the formation of distinct weathering fissures.
3. A quantitative relationship between chemical weathering indices and the mechanical parameters $\:{\phi\:}^{{\prime\:}}$ and *CSR*_20_ of WGS was established. The results indicate a strong linear correlation between the chemical weathering indices and the mechanical properties of WGS, with the coefficient of determination reaching as high as 99%. However, there are instances where certain parameters exhibit a relatively scattered fitting effect, the coefficient of determination is only 60%.

In short, extensive data collection and increased experimental groups will be conducted to further refine the relevant research. To achieve the prediction of macroscopic mechanical indicators using microscopic parameters.

## Data Availability

The datasets generated during and analysed during the current study are available from the corresponding author on reasonable request.
